# Chloroplast genome of tropical and sub-tropical fruit tree *Syzygium samarangense* (Myrtaceae)

**DOI:** 10.1080/23802359.2018.1501296

**Published:** 2018-08-13

**Authors:** Jin Liu, Shu-Bang Ni, Cheng Zheng, Chao Shi, Ying-Feng Niu

**Affiliations:** a Yunnan Institute of Tropical Crops, Jinghong, China;; b Kunming Institute of Botany, Chinese Academy of Sciences, Kunming, China

**Keywords:** Chloroplast genome, *Syzygium samarangense*, Myrtaceae

## Abstract

*Syzygium samarangense* is an important fruit of Myrtaceae family and commercially planted in tropical areas of the world. Chloroplast genome sequences play a significant role in the development of molecular markers in plant phylogenetic and population genetic studies. In this study, we report the complete chloroplast genome sequence of *S. samarangense* for the first time (accession number of MH371141). The chloroplast genome is 159,109 bp long and includes 113 genes. Its LSC, SSC and IR regions are 88,533, 18,882, and 25,847 bp long, respectively. Phylogenetic tree analysis exhibited that *S. samarangense* was clustered with other Myrtaceae species with 100% bootstrap values.

The wax apple (*S. samarangense* (Blume) Merr. and L.M. Perry), which belongs to the family Myrtaceae, is native to the Malaysian Archipelago and to the Andaman and Nicobar islands where the trees grow in coastal rainforests (Morton 1987). Other species with similar fruits for fresh consumption that are commercially important are the water apple *S*. *aqueum*, the rose apple *S. jambos*, and the Malay apple *S. malaccense* (Nakasone and Pall 1988). All these species have spread throughout the tropical areas of the world. The fruit of the wax apple is larger and sweeter (Shü and Paull 2008) and since it is most delicious, it has been commercially planted in many countries, such as Taiwan, Thailand, Indonesia and Malaysia (Shü et al. 2008). Research usually focuses on *S. samarangense* since it is the only species commercially planted on a large scale among the four important freshly consumed species.

In this study, we report the complete chloroplast genome of *S. samarangense* in Myrtaceae. DNA material was isolated from mature leaves of a *S. samarangense* plant cultivated in the plant garden of Yunnan Institute of Tropical Crops (YITC), Jinghong, China by using DNeasy Plant Mini Kit (QIAGEN, Germany). A specimen of this tree and the isolated DNA were stored in YITC. About 10 μg isolated DNA was sent to BGI, Shenzhen for library construction and genome sequencing on the Illumina Hiseq 2000 Platform. After genome sequencing, a total of 4.3 Gbp reads in fastq format were obtained and subjected to chloroplast genome assembly. The complete chloroplast genome was annotated with Dual Organelle GenoMe Annotator (DOGMA; Wyman et al. 2004) and submitted to the Genbank under the accession number of MH371141. Our assembly of the *S. samarangense* resulted in a final sequence of 159,109 bp in length with no gap. The overall A–T content of the chloroplast genome was 61.1%. This chloroplast genome included a typical quadripartite structure with the Large Single Copy (LSC), Small Single Copy (SSC), and Inverted Repeat (IR) regions of 88,533, 18,882, and 25,847 bp long, respectively. Genome annotation showed 113 full length genes including 79 protein-coding genes, 30 tRNA genes and 4 rRNA genes. The genome organization, gene content and gene relative positions were almost identical to the previously reported Myrtaceae chloroplast genomes. To validate the phylogenetic relationships of *S. samarangense* in the Myrtaceae, we constructed a maximum likelihood tree using 9 Myrtaceae taxa. The whole chloroplast genome sequences were aligned with the MAFFT (Katoh and Standley 2013) and the phylogenetic analysis was performed on selected taxa using RAxML (Stamatakis et al. 2008). The resulting tree shows that *S. samarangense* forms a clade with the species of *C. cumini* with a 100% bootstrap value (Figure 1).

**Figure 1.**
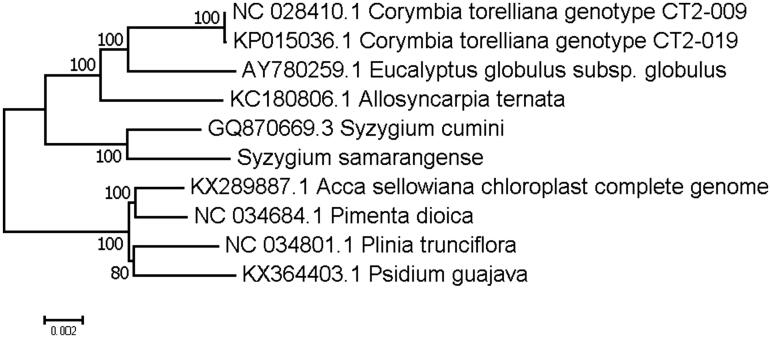
Maximum-likelihood (ML) phylogenetic tree of *S. samarangense* in Myrtaceae. Number above each node indicates the ML bootstrap support values.
